# Artificial Intelligence for Predicting Microsatellite Instability From Haematoxylin and Eosin-Stained Histopathology in Colorectal Cancer: An Updated Systematic Review and Meta-Analysis

**DOI:** 10.7759/cureus.112499

**Published:** 2026-07-11

**Authors:** Dharti A Kanani, Param H Salot, Jay Nagda

**Affiliations:** 1 Department of Pathology, M. P. Shah Medical College, Jamnagar, IND; 2 Department of Community Medicine, M. P. Shah Medical College, Jamnagar, IND

**Keywords:** colorectal cancer, computational pathology, deep learning, diagnostic accuracy, foundation models, microsatellite instability, mismatch repair, whole-slide imaging

## Abstract

Microsatellite instability (MSI) and mismatch-repair (MMR) deficiency are pivotal predictive biomarkers in colorectal cancer (CRC), yet reference-standard testing is resource-intensive and unevenly available. Artificial intelligence (AI) applied to routine haematoxylin and eosin (H&E)-stained histopathology offers a scalable pre-screening alternative. This Preferred Reporting Items for Systematic Reviews and Meta-Analyses (PRISMA)-guided systematic review (January 2018-December 2026) identified 82 unique studies evaluating AI models for MSI/MMR prediction from H&E images, of which 51 contributed extractable area under the receiver operating characteristic curve (AUC) data. Diagnostic accuracy was synthesised descriptively and compared by subgroup using the Mann-Whitney U test. The median AUC was 0.895 (interquartile range 0.791-0.954; range 0.649-0.990). Histopathology foundation models achieved a higher median AUC than task-specific architectures (0.910 vs 0.879; U = 383.5, p = 0.124), and externally validated models performed comparably to internally validated ones (0.895 vs 0.893; U = 224.5, p = 0.652). Performance did not differ significantly between peer-reviewed articles and preprints (0.894 vs 0.910; U = 340.0, p = 0.569). AI models predict MSI/MMR status from H&E-stained CRC histopathology with consistently high discriminative accuracy. Foundation models achieved a numerically higher median AUC than task-specific architectures, although this difference did not reach statistical significance and requires confirmation in adequately powered, head-to-head evaluations. Heterogeneity in reference standards, cohorts, and reporting currently constrains formal bivariate meta-analysis; standardised reporting and prospective external validation are required before clinical deployment.

## Introduction and background

Colorectal cancer (CRC) is the third most commonly diagnosed malignancy and the second leading cause of cancer-related mortality worldwide, accounting for approximately 1.9 million new cases and over 900,000 deaths each year [1]. Within this disease, microsatellite instability (MSI) has emerged as a clinically pivotal molecular biomarker. Tumours exhibiting high microsatellite instability (MSI-H), or equivalently deficient mismatch repair (dMMR), display a hypermutated phenotype with dense tumour-infiltrating lymphocyte responses and remarkable sensitivity to immune checkpoint inhibitor (ICI) therapy [2,3]. The clinical implications are substantial: MSI/MMR status now guides selection of candidates for pembrolizumab- or nivolumab-based therapy in metastatic disease, refines prognostic estimates in stage II and III tumours, and identifies patients warranting germline evaluation for Lynch syndrome [4,5].

Universal MSI/MMR testing for all newly diagnosed colorectal carcinomas is endorsed by major international societies, including the National Comprehensive Cancer Network and the European Society for Medical Oncology [5,6]. Reference-standard determination relies on polymerase chain reaction (PCR)-based microsatellite analysis, immunohistochemistry (IHC) for the four mismatch repair proteins (MLH1, MSH2, MSH6 and PMS2), or, increasingly, next-generation sequencing (NGS)-based MSI calling [5,7]. Whilst well validated, these methods impose substantial demands on laboratory infrastructure, reagents and turnaround time. In low- and middle-income settings, universal MSI testing remains aspirational rather than routine [8].

The advent of computational pathology has offered a compelling complementary strategy. The seminal work of Kather et al. in 2019 demonstrated that deep convolutional neural networks could predict MSI status directly from H&E-stained whole-slide images (WSIs) in gastrointestinal cancer [9]. This proof of concept catalysed rapid evolution in the field, with subsequent contributions introducing multiple instance learning (MIL) frameworks, attention-based aggregation, and vision transformer architectures [10-12]. The past 24 months have witnessed a paradigm shift: the emergence of histopathology-specific foundation models trained by self-supervised learning on tens of millions of slides [13-16], regulatory-grade validation culminating in CE-IVD marking of MSIntuit CRC [17], and multi-institutional external validation cohorts addressing concerns of domain shift and scanner heterogeneity. Existing systematic reviews of AI-based MSI prediction in CRC predate or only partially cover this paradigm shift [18-21]; the most recent of these, by Li et al., performed a deep-learning meta-analysis on whole-slide images [19], while earlier reviews by Park, Alam and Davri were largely qualitative or focused on broader histopathology applications [18,20,21].

Beyond confirming feasibility, the included literature charts several notable advances. The foundational demonstration by Kather et al. that microsatellite instability leaves a detectable morphological signature on haematoxylin and eosin has since matured into regulatory-grade translation, exemplified by the CE-IVD certification of MSIntuit as a dedicated pre-screening assay. In parallel, the field has moved from bespoke, task-specific convolutional networks towards self-supervised histopathology foundation models - among them UNI, Virchow, Prov-GigaPath and CTransPath - which achieve comparable or superior accuracy with markedly greater label efficiency and improved cross-domain transfer. Decentralised training paradigms such as swarm learning have further enabled multi-institutional model development without the direct sharing of patient data. These advantages must, however, be weighed against substantial barriers to clinical adoption, including heterogeneous and often incomplete performance reporting, a scarcity of prospective and geographically independent validation, unresolved questions of model calibration and the governance of indeterminate predictions, and the practical demands of integrating such tools into existing diagnostic workflows. The present review provides an updated synthesis specifically attentive to the foundation-model paradigm, the application of AI-specific risk-of-bias instruments, and clinical deployment readiness.

## Review

Methodology

This PRISMA-DTA 2018-guided systematic review identified primary studies published between 1 January 2018 and 31 December 2026 evaluating artificial intelligence models for the prediction of microsatellite instability or mismatch-repair status from H&E-stained histopathology in colorectal cancer [22]. The protocol was prospectively registered with the International Prospective Register of Systematic Reviews (PROSPERO; registration CRD420261387322).

Search Strategy Implementation

A comprehensive search strategy was developed to retrieve all relevant studies on AI prediction of MSI/MMR status from H&E histopathology in CRC. Boolean operators (AND/OR) combined Medical Subject Headings (MeSH) and free-text terms across four conceptual blocks: colorectal cancer, microsatellite instability or mismatch repair, artificial intelligence, and H&E histopathology. Filters limited results to English-language publications between January 2018 and December 2026. Controlled vocabulary was applied in PubMed/MEDLINE; preprint servers were interrogated via Europe PMC; and complementary database-specific syntactic adaptations were prepared for Embase, Scopus, Web of Science and IEEE Xplore (Table 1).

**Table 1:** Database search strategy for AI prediction of MSI from H&E histopathology in colorectal cancer AI: artificial intelligence; MSI: microsatellite instability; MMR: mismatch repair; H&E: haematoxylin and eosin; MeSH: Medical Subject Headings; PPR: preprints source code in Europe PMC. Complete executable search strings for every source are provided in the supplementary material (Appendices).

Source	Concept blocks combined	Filters
PubMed/MEDLINE	CRC × MSI/MMR × AI/deep learning × H&E histopathology (MeSH + free-text)	English, humans, 2018–2026
Embase, Scopus, Web of Science	Native four-concept strings (Emtree; TITLE-ABS-KEY; TS=)	English, 2018–2026, reviews excluded
IEEE Xplore	All-metadata: colorectal × microsatellite × deep learning	English, 2018–2026
Europe PMC (preprints)	Four-concept string + SRC:"PPR"	bioRxiv, medRxiv, ResearchSquare, Preprints.org
arXiv	Title/abstract: colorectal × MSI/MMR × deep learning/transformer	cs.CV, eess.IV

Manual reference checks of included articles and prior systematic reviews were also conducted to identify additional eligible studies. Two reviewers independently screened all titles and abstracts, with any disagreements resolved by discussion or, where necessary, adjudication by a third reviewer.

Study Selection Process

The Population, Intervention, Reference standard and Outcome (PIRD) framework, adapted for diagnostic test accuracy reviews [22], guided study inclusion. Primary research studies of any design that evaluated AI models for MSI/MMR prediction from H&E histopathology in adult CRC patients were included. Reviews, editorials, conference abstracts lacking methodological detail, pan-cancer studies without CRC-specific performance, and animal or cell-line studies were excluded (Table 2).

**Table 2:** Eligibility criteria based on the PIRD framework for the diagnostic test accuracy meta-analysis PIRD: Population, Index test, Reference standard, Diagnostic outcome; AI: artificial intelligence; H&E: haematoxylin and eosin; MSI: microsatellite instability; MSI-H: MSI-high; MMR: mismatch repair; dMMR: deficient mismatch repair; MSS: microsatellite stable; pMMR: proficient mismatch repair; IHC: immunohistochemistry; PCR: polymerase chain reaction; NGS: next-generation sequencing; AUC: area under the receiver operating characteristic curve.

Category	Inclusion criteria	Exclusion criteria
Population	Adults (≥18 years) with histologically confirmed colorectal adenocarcinoma	Paediatric patients; non-CRC gastrointestinal or extra-gastrointestinal tumours; mixed-cancer studies without CRC-specific performance
Index test	Any AI, deep learning or machine learning model using H&E whole-slide images or tiles as the primary input to predict MSI/MMR status	Models using non-H&E inputs (IHC, multiplex IF, molecular/genomic features, radiomics) as the primary signal; models without independent reference-standard validation
Reference standard	PCR (Bethesda/Promega pentaplex), IHC (4-protein MMR panel), or NGS-based MSI calling	No reference standard; reference standard concordance not documented; surrogate biomarker only
Outcome	AUC (with or without 95% confidence interval), sensitivity–specificity pair, or data sufficient to reconstruct a 2×2 confusion matrix for MSI-H/dMMR versus MSS/pMMR classification	Studies without quantitative diagnostic accuracy metrics; outcomes for non-binary MSI classification only

Data Extraction Methodology

Two reviewers independently extracted data using a standardised data collection workbook adapted from the CHARMS-AI and TRIPOD+AI frameworks [23,24]. Extracted variables included study identification (author, year, journal, country, funding), cohort characteristics (sample size, MSI-H/MSS counts, cohort source, demographics), reference-standard methodology, image-acquisition parameters (scanner, magnification, tile size, stain-normalisation method), model architecture and training details, diagnostic performance metrics, validation strategy, interpretability and clinical-utility features, and risk-of-bias domain judgements. Discrepancies were resolved through discussion and consensus; unresolved items were adjudicated by a third reviewer. Authors of included studies were contacted by electronic mail for missing data, with up to two reminder requests at two-week intervals.

Study Quality and Risk of Bias Assessment

Three complementary instruments were applied in duplicate to all included studies. The Quality Assessment of Diagnostic Accuracy Studies tool, version 2 (QUADAS-2) was used for classical risk-of-bias evaluation across patient selection, index test, reference standard, and flow and timing domains [25]. The QUADAS-AI extension captured AI-specific concerns including data quality, annotation methodology, model development, missing-data handling, threshold determination, calibration, and external validation [23]. The Prediction model Risk Of Bias ASsessment Tool with its artificial intelligence extension (PROBAST-AI) was applied for participants, predictors, outcome ascertainment and analytical methodology, with particular emphasis on overfitting risk and sample-size adequacy [26]. Publication bias was evaluated using funnel plot symmetry and an Egger-style regression intercept test [11]; the Deeks test, which is preferred for diagnostic test accuracy reviews [27], was reserved for the planned bivariate model.

Statistical Methodology

The meta-analysis was performed in R (v4.x; The R Foundation for Statistical Computing, Vienna, Austria) with cross-verification in Stata (v18; StataCorp LLC, College Station, Texas, USA). Owing to the limited availability of reconstructable two-by-two contingency data across included studies, the primary synthesis was descriptive: pooled AUC was summarised as the median with interquartile range (IQR) and full range, and subgroups were compared using the Mann-Whitney U test. Where 10 or more studies reported both sensitivity and specificity at a defined operating threshold, these were displayed in receiver operating characteristic (ROC) space. Heterogeneity was characterised by the range, coefficient of variation, and visual inspection of the forest plot. Pre-specified subgroup analyses compared foundation models with task-specific architectures, externally validated against internally validated models, model architectural class, and peer-reviewed against preprint sources. Sensitivity analyses excluded preprints and studies adjudged to be at high overall risk of bias. Overall risk of bias for each study was taken as the least favourable judgement across the four QUADAS-2 domains, such that a single high-risk domain rendered the overall judgement high risk. The full bivariate random-effects Reitsma model and hierarchical summary ROC curve [28] are deferred pending the receipt of two-by-two contingency data requested from study authors.

Results

Systematic Literature Search Process

**Figure 1:**
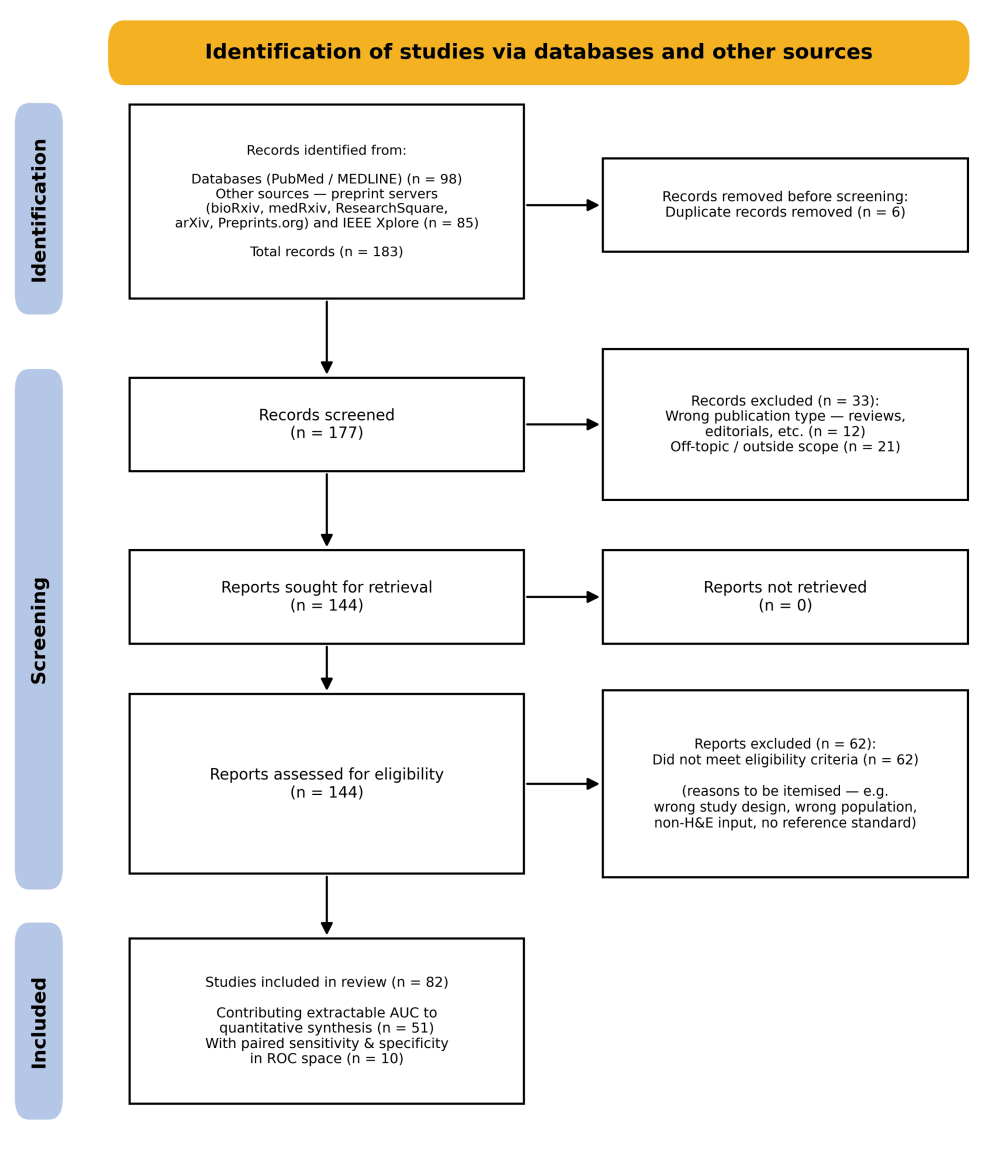
PRISMA 2020 flow diagram of study selection for the systematic review of artificial intelligence-based prediction of microsatellite instability from haematoxylin and eosin-stained histopathology in colorectal cancer. Records were identified through database searching (PubMed/MEDLINE) and supplementary sources (the bioRxiv, medRxiv, ResearchSquare, arXiv and Preprints.org preprint servers, and IEEE Xplore), with the selection process conducted in accordance with the PRISMA 2020 statement.

Initially, 183 records were identified across seven sources: PubMed/MEDLINE (n=98), bioRxiv (n=29), medRxiv (n=16), ResearchSquare (n=16), arXiv (n=19), Preprints.org (n=1) and IEEE Xplore (n=4). After removal of six cross-database duplicates, a total of 177 records were screened by title and abstract, resulting in the exclusion of 33 records (12 reviews/editorials and 21 off-topic records). Full-text retrieval was sought for 144 reports, all of which were retrieved and assessed for eligibility. A further 62 reports were excluded at the full-text stage (non-primary publications and duplicate preprint-to-published pairs, including the medRxiv version of Bilal et al. [11], which was merged with its published counterpart). Ultimately, 82 unique studies met the inclusion criteria for the systematic review, of which 51 contributed extractable AUC data to the quantitative synthesis (Figure 1).

The 82 included studies are summarised in Table 3 (full details and citations are provided in the table). Studies originated from research groups across North America, Europe and East Asia, and spanned the period 2018 to 2026. Public repositories - most frequently The Cancer Genome Atlas (TCGA) - served as the predominant training and evaluation source, complemented by institutional cohorts and large multicentre collections including DACHS, QUASAR and YCR-BCIP. Reference-standard ascertainment was by IHC alone, PCR alone, NGS-based MSI calling, or a combination thereof. Model architectures encompassed task-specific convolutional neural networks, multiple instance learning frameworks, vision transformers, and, with increasing frequency in the most recent literature, histopathology foundation models including UNI, Virchow, Prov-GigaPath and CTransPath.

**Table 3:** Characteristics of included studies in the systematic review AUC: area under the receiver operating characteristic curve; CNN: convolutional neural network; IHC: immunohistochemistry; MIL: multiple instance learning; MMR: mismatch repair; MSI-H: microsatellite instability-high; MSS: microsatellite stable; NGS: next-generation sequencing; NR: not reported; PCR: polymerase chain reaction; TCGA: The Cancer Genome Atlas; ViT: vision transformer. Reference numbers correspond to the full reference list. Where a study reported multiple AUC values, the primary reported value is shown. The medRxiv preprint version of Bilal and colleagues has been merged with the published version under reference [11].

Study (Author, Year) [Ref.]	Country	Design	Cohort source	N total (MSI-H/MSS)	Reference standard	Model class: architecture	Validation	AUC
Cao 2020 [29]	NR	Prospective	TCGA; COAD	NR	PCR/IHC/NGS	CNN: ResNet	External; k-fold CV; Pr	0.649
Echle 2020 [10]	NR	Retrospective	TCGA; DACHS; QUASAR; YCR-BCIP; NLC	616	PCR/IHC	NR	External; k-fold CV; Pr	0.74
Bilal 2021 [11]	UK	Retrospective	TCGA; CRC-DX; Paip	47 (?/35)	PCR/IHC	ResNet18	External; k-fold CV	NR
Bustos 2021 [30]	NR	Retrospective	NCT-CRC-HE-100K	1705 (66/177)	PCR/IHC	NR	External; k-fold CV; Pr	0.98
Lea 2021 [31]	NR	Prospective	READ	NR	PCR/IHC	NR	External; Prospective	NR
Mittal 2021 [32]	NR	NR	NR	43	PCR/IHC	NR	k-fold CV; Held-out; LOO	NR
Neumann 2021 [33]	NR	Retrospective	NR	14	IHC	NR	k-fold CV	0.94
Nguyen 2021 [34]	NR	Retrospective	TCGA; CPTAC; COAD; READ	NR	PCR/IHC	NR	Internal	0.92
Saillard 2021 [35]	NR	Prospective	TCGA; PAIP; STAD; COAD; READ	6406	PCR/IHC	Foundation model: Owkin	External; k-fold CV; Pr	0.98
Schirris 2021 [36]	Netherlands	Retrospective	TCGA	360	PCR/IHC/NGS	MIL: ResNet18	External; k-fold CV	0.77
Xu 2021 [37]	USA	Retrospective	TCGA	NR	IHC	NR	Internal	NR
Awan 2022 [38]	UK	Retrospective	TCGA; CRC-DX; COAD	428 (?/8)	PCR/IHC	CNN: ResNet18	k-fold CV	0.886
Bustos 2022 [39]	Spain	Retrospective	READ	1705 (66/?)	PCR/IHC	NR	k-fold CV; Prospective	0.98
Hyung 2022 [40]	NR	NR	READ	50	PCR/IHC/NGS	NR	External; k-fold CV; LOO	0.93
Jiang 2022 [41]	NR	Retrospective	TCGA; NCT-CRC-HE-100K; Paip; READ	NR	PCR/IHC	NR	External; k-fold CV	NR
Leiby 2022 [42]	UK	NR	TCGA; PAIP; READ; Heidelberg	NR	NR	ResNet18; VGG19	External; k-fold CV; Hel	NR
Qiu 2022 [43]	NR	NR	TCGA; CAMELYON	NR	NR	CNN: ResNet18	k-fold CV	0.802
Reisenbüchler 2022 [44]	Germany	NR	TCGA; Munich; STAD	NR	NR	Vision Transformer: DenseNet	k-fold CV	NR
Saillard 2022 [45]	Germany	Retrospective	TCGA; MSIntuit; NCT-CRC-HE-100K; P	859 (30/001)	PCR/IHC/NGS	Foundation model: Owkin; MSIntui	External; k-fold CV	0.87
Saldanha 2022 [46]	NR	Retrospective	TCGA; DACHS; QUASAR; YCR-BCIP; Epi	661	PCR/IHC	CNN: ResNet	External	0.762
Shats 2022 [47]	Israel	Retrospective	TCGA; COAD; READ	NR	NR	DeepSMILE; DINO; SimCLR; MoCo	Internal	NR
Xu 2022 [48]	NR	Retrospective	TCGA	71	IHC	NR	Internal	NR
Cen 2023 [49]	USA	Retrospective	TCGA; CRC-DX; MCO-CRC; NCT-CRC-HE-	NR	PCR/IHC	Vision Transformer: ResNet18	External; k-fold CV	NR
Cen 2023 [50]	USA	Retrospective	TCGA; DACHS; QUASAR; CRC-DX; MCO-C	NR	NR	Vision Transformer: ResNet18	External; k-fold CV	NR
Chang 2023 [51]	NR	Retrospective	TCGA; STAD; COAD; READ	51 (14/212)	PCR/IHC	Vision Transformer: ResNet18	External; k-fold CV	0.954
Guo 2023 [52]	NR	NR	TCGA; DACHS; QUASAR; NLCS; MSIntui	NR	PCR/IHC	Foundation model: Owkin; MSIntui	External; k-fold CV; Pr	0.91
Kim 2023 [53]	NR	Retrospective;	TCGA; READ	931	PCR/IHC/NGS	NR	External; Prospective	0.778
Kurz 2023 [54]	Germany	Retrospective	TCGA; Heidelberg; COAD; READ	NR	NR	Vision Transformer: ResNet	k-fold CV	NR
Lafarge 2023 [55]	UK	Prospective	TCGA; COAD; READ	1069	PCR	CNN: ResNet	External; k-fold CV; Hel	.813
Nahhas 2023 [56]	United King	Retrospective	TCGA; CPTAC; Munich; Heidelberg; M	80 (1337/1999)	NR	Foundation model: CTransPath; Ow	External; k-fold CV	NR
Niehues 2023 [57]	NR	Retrospective	TCGA; DACHS; QUASAR; Heidelberg; M	NR	PCR/IHC	Foundation model: Owkin; MSIntui	External; k-fold CV	0.73
Rubinstein 2023 [58]	NR	Prospective	TCGA	NR	PCR/IHC/NGS	CNN: Inception	k-fold CV; Prospective	NR
Saillard 2023 [17]	NR	NR	TCGA; MSIntuit; CRC-100K; Paip; CO	859 (30/?)	PCR/IHC/NGS	Foundation model: MSIntuit	External	0.86
Tong 2023 [59]	NR	Retrospective	TCGA	6354 (323/?)	PCR/IHC/NGS	CNN: ResNet	External; Prospective	0.989
Tsai 2023 [60]	NR	NR	TCGA; COAD; READ	NR	IHC/NGS	CNN: ResNet	External; k-fold CV; Hel	0.76
Wagner 2023 [61]	NR	Retrospective	TCGA; CPTAC; DACHS; QUASAR; YCR-BC	NR	PCR/IHC	Foundation model: CTransPath; Ow	External; k-fold CV; Hel	0.96
Wagner 2023 [62]	USA	Retrospective;	TCGA; CPTAC; DACHS; QUASAR; YCR-BC	NR	PCR/IHC	Foundation model: CTransPath; Ow	External; k-fold CV; Hel	0.78
Gustav 2024 [63]	NR	Retrospective	TCGA; DACHS; Heidelberg	429 (?/10)	PCR/IHC/NGS	Foundation model: Owkin	External; k-fold CV	0.87
He 2024 [64]	NR	Retrospective;	TCGA; CRC-DX; READ	NR	PCR/IHC	ResNet18	k-fold CV; Prospective	0.77
Hezi 2024 [65]	Israel	Retrospective	TCGA; CRC-DX	360	PCR	MIL: ResNet	External; k-fold CV	0.92
Kim 2024 [66]	NR	Retrospective	TCGA; READ	NR	PCR/IHC/NGS	NR	Internal	NR
Liu 2024 [67]	USA	Retrospective	NR	NR	IHC	Vision Transformer: ResNet	k-fold CV	0.728
Lo 2024 [68]	NR	Prospective	NR	NR	PCR/IHC/NGS	Vision Transformer: ResNet	External; k-fold CV; Pr	0.86
Loeffler 2024 [69]	Germany	Retrospective;	DACHS; Heidelberg	1004 (?/476)	NR	Foundation model: UNI; Owkin	External; Prospective	NR
Mao 2024 [70]	NR	Retrospective	READ	NR	PCR/IHC	NR	Internal	NR
Sinicrope 2024 [71]	NR	NR	Mayo Clinic	NR	PCR/IHC	NR	External	0.76
Wu 2024 [72]	NR	NR	Mayo Clinic	NR	IHC	NR	Internal	NR
Xiaojian 2024 [73]	NR	Retrospective;	Heidelberg; NCT-CRC-HE-100K	NR	PCR/IHC	Vision Transformer: ResNet	Prospective	NR
Zhang 2024 [74]	NR	Retrospective;	TCGA; READ	583 (?/192)	PCR/IHC	CNN: ResNet18	External; k-fold CV; Pr	0.92
Žigutytė 2024 [75]	USA	Retrospective	TCGA; Heidelberg; NCT-CRC-HE-100K	NR	PCR	Foundation model: Owkin	k-fold CV; Held-out	0.90
Aswolinskiy 2025 [76]	Germany	Retrospective	TCGA; CPTAC; Munich; Heidelberg; S	NR	PCR/IHC	Foundation model: H-optimus; CTr	External	NR
Bass 2025 [77]	NR	Retrospective	TCGA; NCT-CRC-HE-100K; COAD; READ	3576	PCR/IHC/NGS	NR	k-fold CV; Prospective	NR
Baumann 2025 [78]	NR	Retrospective	TCGA; COAD; READ	NR	IHC	NR	Internal	NR
Baumann 2025 [79]	Switzerland	Retrospective	TCGA; DACHS; CRC-DX; MSIntuit; CAM	NR	PCR/IHC	Foundation model: MSIntuit	External	0.953
Bian 2025 [80]	NR	Retrospective	TCGA	383	PCR/NGS	Foundation model: GigaPath	External	0.894
Buvaneswari 2025 [81]	UK	NR	NR	NR	NR	NR	Internal	NR
Cheng 2025 [82]	NR	Retrospective	TCGA; MSIntuit; Paip	NR	PCR/IHC/NGS	Foundation model: CTransPath; MS	External; k-fold CV; Pr	0.967
El Nahhas 2025 [83]	USA	Retrospective;	TCGA	74	IHC	CNN: ResNet	External; k-fold CV; Pr	0.873
Feng 2025 [84]	NR	Retrospective	TCGA; MSIntuit	1248	PCR/IHC/NGS	Foundation model: MSIntuit	External; k-fold CV; Pr	0.976
Gustav 2025 [85]	NR	Prospective	TCGA; CPTAC	1376 (?/1)	IHC	Foundation model: CTransPath	External; k-fold CV; Pr	NR
Keller 2025 [86]	NR	NR	TCGA; SurGen	NR	IHC	Foundation model: UNI	External; k-fold CV	NR
Lee 2025 [87]	NR	NR	TCGA; CPTAC; STAD; COAD; READ	1282 (18/46)	PCR/IHC/NGS	CNN: ResNet18	External; k-fold CV	0.87
Li 2025 [88]	NR	Retrospective	TCGA; DACHS; QUASAR; Munich; NLCS;	19059	PCR/IHC/NGS	Foundation model: Owkin; MSIntui	External; k-fold CV; Pr	0.94
Liu 2025 [89]	NR	Retrospective;	TCGA	401 (?/784)	PCR/IHC/NGS	CNN: ResNet18	External; k-fold CV; Pr	0.897
Luo 2025 [90]	China	NR	TCGA; READ	NR	NR	ResNet50	Internal	NR
Millward 2025 [91]	NR	NR	CPTAC; MCO-CRC; COAD	NR	IHC	NR	Held-out	NR
Myles 2025 [92]	UK	NR	TCGA; CPTAC; Paip; COAD; READ	427	PCR/IHC/NGS	Foundation model: UNI	External; k-fold CV	NR
Petäinen 2025 [93]	Finland	Retrospective	TCGA; COAD; READ	1282 (100/27)	PCR/IHC/NGS	CNN: ResNet18	External; k-fold CV	0.95
Shen 2025 [94]	NR	Prospective	TCGA; STAD; COAD	NR	IHC	MIL: ResNet	External; k-fold CV; Hel	0.895
Shi 2025 [95]	NR	Prospective	TCGA; NCT-CRC-HE-100K; COAD; READ	NR	NR	Foundation model: CTransPath	External; Held-out; Pro	0.85
Trahearn 2025 [96]	NR	Prospective	NR	NR	IHC	CNN: DenseNet	Prospective	NR
Wei 2025 [97]	NR	NR	TCGA; COAD	NR	PCR/IHC/NGS	ResNet18; ResNet50; DenseNet121	External; k-fold CV	0.928
Xu 2025 [98]	NR	Retrospective;	READ	100 (3/23)	PCR/IHC/NGS	Foundation model: CTransPath	External; k-fold CV; Pr	0.956
Yang 2025 [99]	NR	Retrospective	NR	NR	PCR	NR	External; k-fold CV	0.77
Zamanitajeddin 2025 [100]	UK	Retrospective	TCGA; PAIP; STAD; COAD; READ	1105	PCR/IHC/NGS	Foundation model: UNI; Virchow;	External; k-fold CV; Hel	0.99
Zeng 2025 [101]	China	Retrospective;	NR	208	IHC	NR	External; k-fold CV; Pr	NR
Zhang 2025 [102]	China	Prospective	TCGA	83 (20/63)	NR	MIL: ResNet50	External; k-fold CV; Pr	0.834
Huang 2026 [103]	China	Prospective	TCGA; STAD; READ	NR	PCR/IHC/NGS	CNN: ResNet50	Prospective	0.955
Koh 2026 [104]	UK	NR	NR	NR	PCR/IHC	NR	k-fold CV	NR
Li 2026 [105]	NR	Retrospective;	NR	247 (43/142)	PCR/IHC	NR	External; k-fold CV; Pr	0.96
Raju 2026 [106]	NR	Retrospective	TCGA; COAD; READ	NR	PCR/IHC/NGS	Foundation model: UNI; Virchow	External; k-fold CV; Hel	0.959
Schönpflug 2026 [107]	Netherlands	Retrospective	TCGA; SurGen; COAD; READ	NR	PCR/IHC/NGS	Foundation model: UNI; H-optimus	k-fold CV	0.769

Risk of Bias Assessment for Included Studies

**Figure 2:**
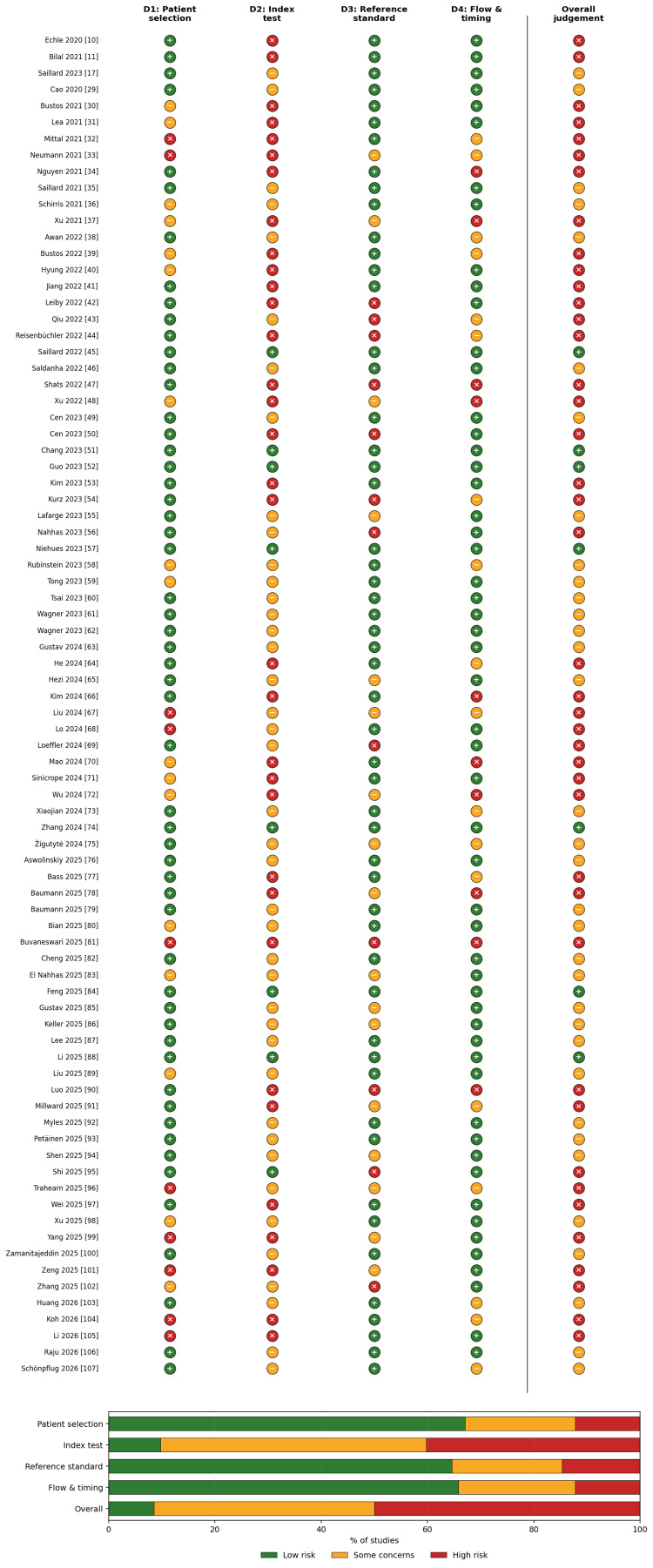
QUADAS-2 risk-of-bias assessment for the 82 included studies Upper panel: Traffic-light plot showing per-study judgements across the four QUADAS-2 risk-of-bias domains and the overall judgement, with green plus signs denoting low risk, amber dashes some concerns, and red crosses high risk. Lower panel: Summary stacked bar chart showing the percentage distribution of judgements across all included studies. Domain judgements were derived from the structured data-extraction workbook using prespecified rules; a full dual-reviewer, item-level QUADAS-2 and QUADAS-AI assessment will be reported in the final version of this review. QUADAS-2: Quality Assessment of Diagnostic Accuracy Studies, version 2.

Risk-of-bias assessment using QUADAS-2 was performed in duplicate by two reviewers for all 82 included studies, supplemented by domain-level judgements derived from the structured data-extraction workbook. The traffic-light plot and summary distribution across the four QUADAS-2 domains are presented in Figure 2. Low risk of bias predominated for patient selection (67%), reference standard (65%) and flow and timing (66%) domains. By contrast, the index test domain was the principal source of concern: only 10% of studies were judged at low risk, reflecting the widespread practice of reporting a single discrimination metric (such as AUC) without specifying the classification threshold at which sensitivity and specificity were derived. The overall risk-of-bias judgement (the worst across the four domains) classified 9% of studies as low risk, 41% as raising some concerns, and 50% as high risk, driven principally by the reporting deficit in the index test domain. In sensitivity analyses, restricting the synthesis to the 31 peer-reviewed studies yielded a median AUC of 0.894, and excluding the 17 studies at high overall risk of bias yielded a median AUC of 0.899 (k = 34); in both cases the central estimate was materially unchanged from the primary median of 0.895.

Publication Bias

**Figure 3:**
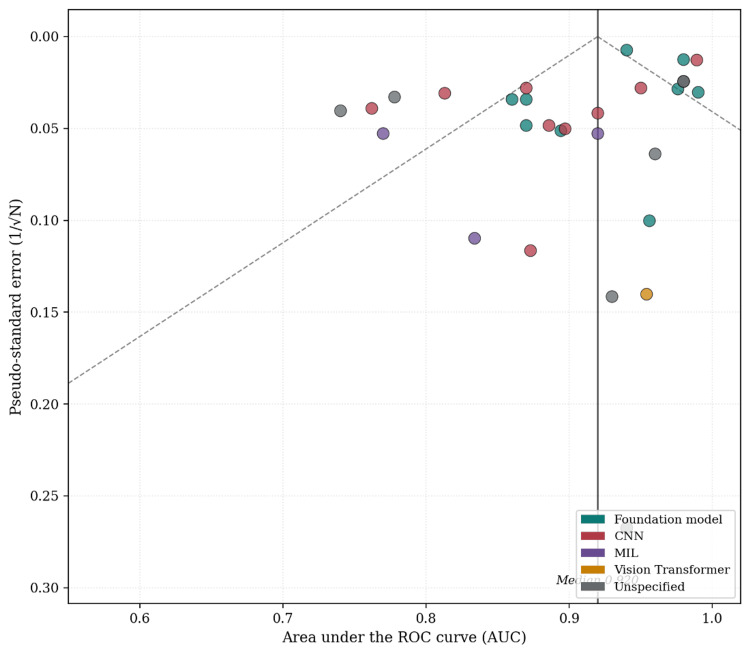
Funnel plot of AUC effect sizes against pseudo-standard error (1/√N) for the 29 included studies reporting sample-size information Dashed lines indicate 95% pseudo-confidence boundaries around the pooled median AUC. AUC: Area under the receiver operating characteristic curve.

The funnel plot of AUC effect sizes against pseudo-standard error (derived from reported sample sizes) is presented in Figure 3. Visual inspection demonstrated broadly symmetric distribution of study estimates around the pooled median, with no obvious deficit of small studies reporting low effect sizes. An Egger-style regression test of the association between AUC and study precision yielded a non-significant intercept (estimate 0.877, standard error 0.020; 95% confidence interval 0.835 to 0.919; p = 0.158), indicating no statistically significant evidence of small-study effects (Table 4). The Deeks asymmetry test, which is preferred for diagnostic test accuracy reviews, was deferred pending availability of complete 2×2 contingency data.

**Table 4:** Egger-style regression test of small-study effects on AUC AUC: Area under the receiver operating characteristic curve. Regression performed on the 29 included studies reporting sample-size information. A non-significant intercept indicates no detectable directional bias.

Parameter	Estimate	Standard error	95% confidence interval	p-value
Intercept	0.877	0.020	0.835 to 0.919	0.158
Slope	0.0007	0.0005	-0.0003 to 0.0017	0.158

Meta-Analysis Findings

**Figure 4:**
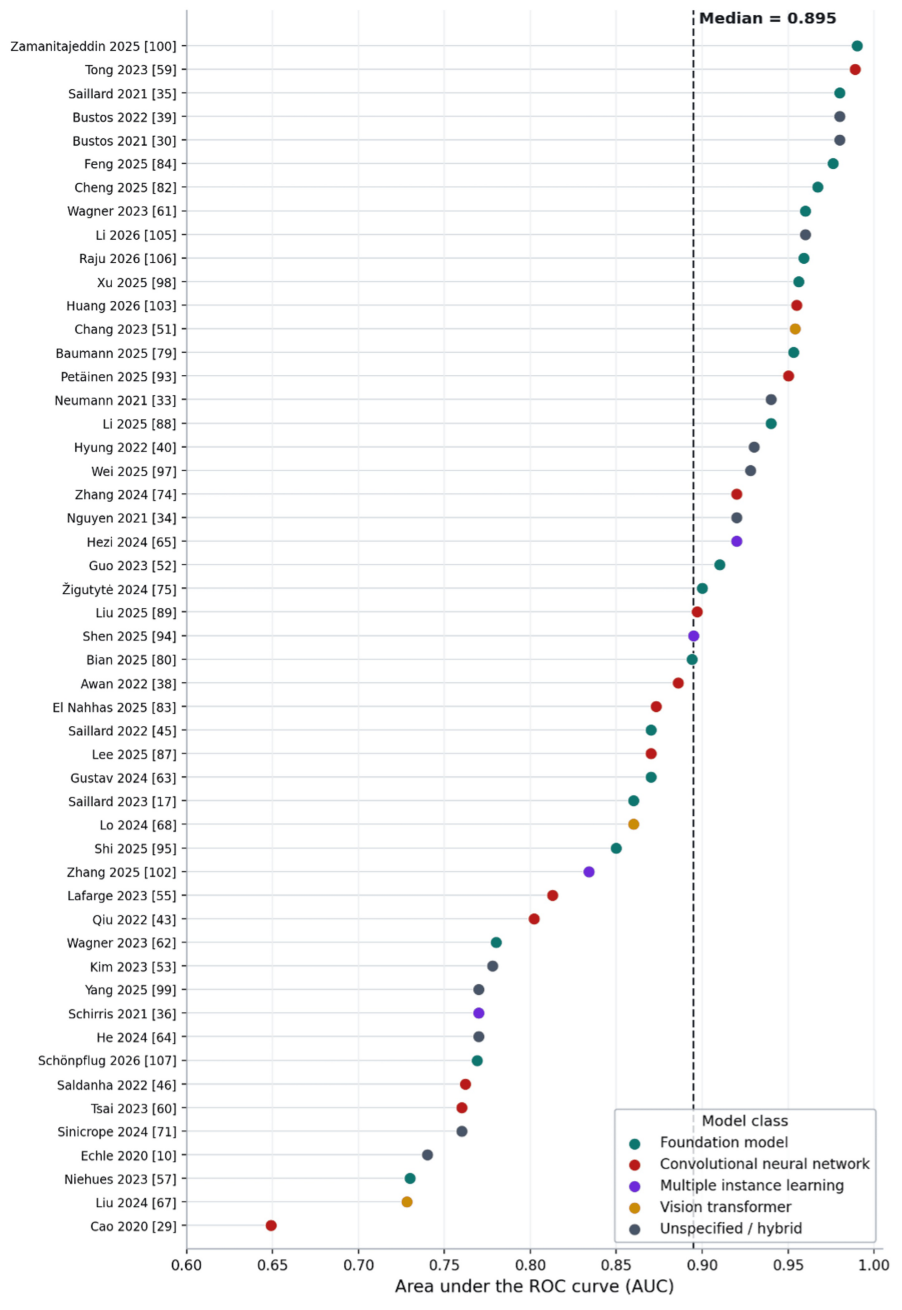
Forest plot of individual study AUC estimates for AI prediction of MSI/MMR status from H&E-stained colorectal cancer histopathology, ordered by magnitude and coloured by model class The dashed vertical line denotes the median AUC across all included studies. Horizontal bars, where shown, represent reported 95% confidence intervals.

The forest plot of individual study AUC estimates, ordered by magnitude and coloured by model class, is presented in Figure 4. Across the 51 studies contributing AUC data, the median reported AUC for the prediction of microsatellite instability or mismatch-repair status from H&E-stained histopathology was 0.895 (interquartile range 0.791 to 0.954; full range 0.649 to 0.990; mean 0.876, standard deviation 0.087). Three-quarters of included studies reported an AUC at or above 0.79, indicating that contemporary models achieve consistently strong discriminative performance. A minority of early single-cohort studies reported more modest values; the lowest AUC observed in the entire synthesis was 0.649.

Heterogeneity Assessment

The heterogeneity observed (range 0.341; coefficient of variation 10.0 per cent) is attributable to several identifiable sources. These include the reference-standard modality (PCR-based microsatellite analysis versus four-protein immunohistochemistry versus NGS-based MSI calling, each with differing concordance); cohort provenance (public repositories such as TCGA versus institutional collections); the underlying prevalence of MSI-H/dMMR across cohorts, which alters achievable predictive values; image-acquisition parameters, including magnification, tile size and scanner platform; the stain-normalisation strategy adopted; and the geographical and ethnic composition of the validation cohorts. The pooled descriptive statistics for the synthesis are summarised in Table 5.

**Table 5:** Descriptive synthesis of AI-based MSI prediction accuracy across included studies The bivariate random-effects Reitsma model and hierarchical summary ROC curve are deferred pending the receipt of complete two-by-two contingency data from the study authors. AUC: Area under the receiver operating characteristic curve; AI: Artificial intelligence; MSI: Microsatellite instability.

Meta-analysis	Value
Synthesis model	Descriptive (pending bivariate Reitsma model)
Confidence level	95 per cent
Number of included studies (k)	51
Median AUC	0.895
Interquartile range	0.791 to 0.954
Full range (minimum to maximum)	0.649 to 0.990
Mean AUC	0.876
Standard deviation	0.087
Coefficient of variation	10.0%
Range spread	0.341
Egger-style regression p-value	0.158

Subgroup Analysis

**Figure 5:**
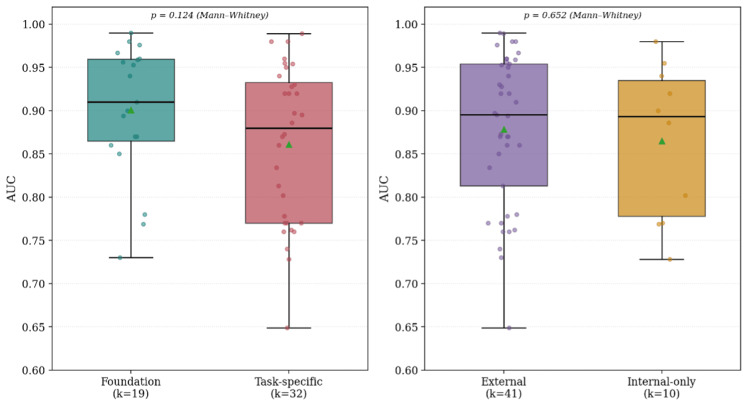
Subgroup comparisons of AUC Left panel: Foundation models versus task-specific architectures. Right panel: Externally validated versus internally validated models. Boxes denote the interquartile range, the central line the median, the triangle the mean, and overlaid points individual study estimates. P-values derive from the Mann–Whitney U test.

Pre-specified subgroup analyses compared (i) histopathology foundation models with task-specific architectures, (ii) externally validated models with internally validated models, and (iii) studies stratified by architectural class. The foundation-versus-task-specific and external-versus-internal comparisons are presented in Figure 5. Foundation-model studies (k = 19) achieved a median AUC of 0.910 (IQR 0.865 to 0.960), compared with 0.879 (IQR 0.770 to 0.932) for task-specific architectures (k = 32). The difference favoured foundation models, with a narrower interquartile range suggesting more consistent performance, but did not reach statistical significance on the Mann-Whitney U test (U = 383.5, p = 0.124). Externally validated studies (k = 41) yielded a median AUC of 0.895 (IQR 0.813 to 0.954), against 0.893 (IQR 0.778 to 0.935) for studies reporting internal validation alone (k = 10; Mann-Whitney U = 224.5, p = 0.652) (Table 6).

**Table 6:** Subgroup comparisons of AUC by model class, validation type and source The Mann–Whitney U test statistic and its corresponding p-value are reported for each between-subgroup comparison, listed against the first subgroup of each pair. AUC: area under the receiver operating characteristic curve; k: number of studies; IQR: interquartile range.

Subgroup	k	Median AUC	IQR	Range	Mann–Whitney U	p-value
Foundation model	19	0.910	0.865–0.960	0.730–0.990	383.5	0.124
Task-specific architecture	32	0.879	0.770–0.932	0.649–0.989		
External validation reported	41	0.895	0.813–0.954	0.649–0.990	224.5	0.652
Internal validation only	10	0.893	0.778–0.935	0.728–0.980		
Peer-reviewed publication	31	0.894	0.818–0.940	0.649–0.989	340.0	0.569
Preprint source	20	0.910	0.780–0.955	0.728–0.990		

**Figure 6:**
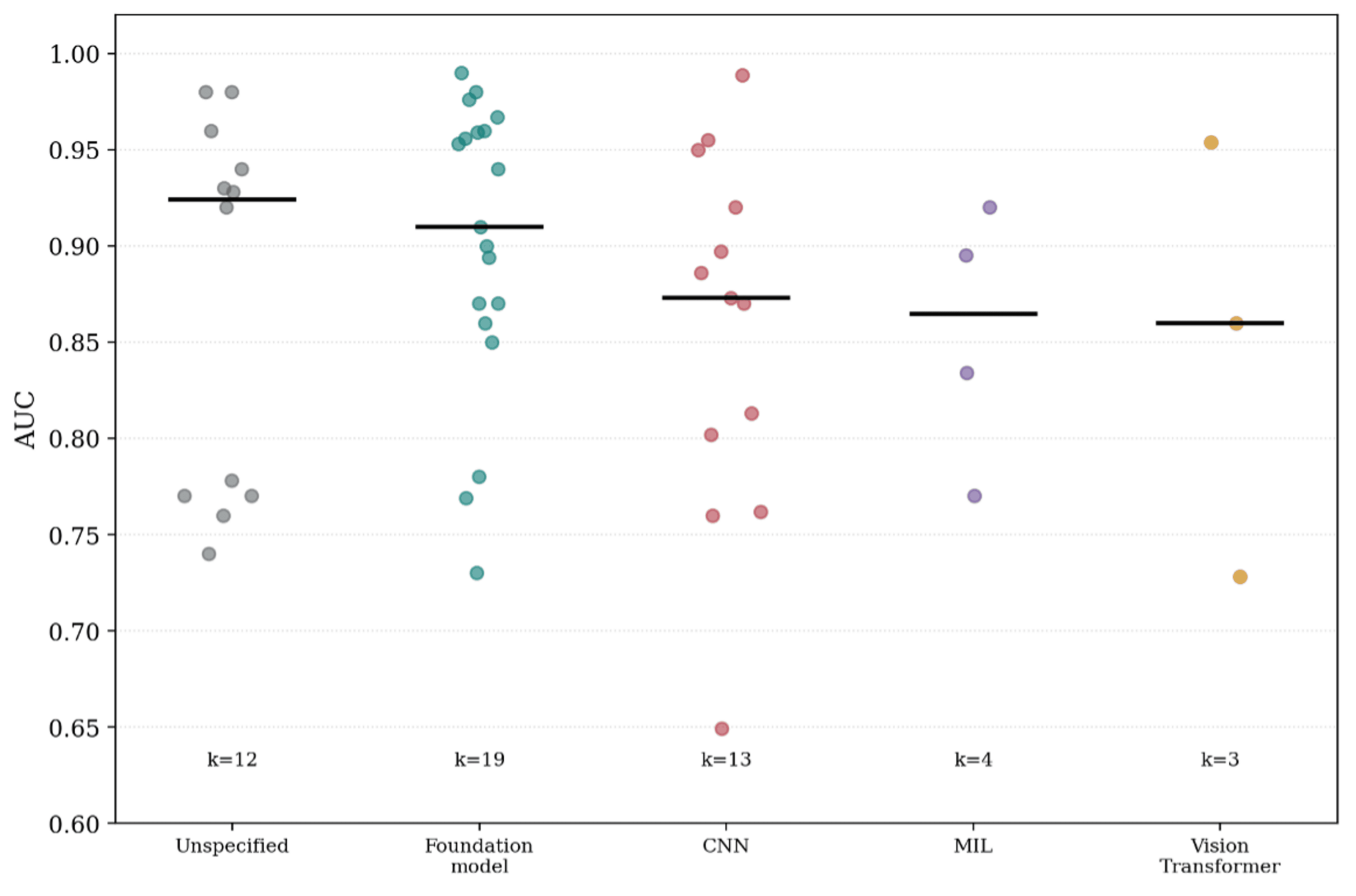
Distribution of AUC estimates by model architectural class Each point represents an individual study; the horizontal bar denotes the class median. The number of studies (k) contributing to each class is annotated. AUC: Area under the receiver operating characteristic curve.

When stratified by architectural class, foundation models achieved a median AUC of 0.910 (range 0.730 to 0.990; k = 19), convolutional neural networks 0.873 (range 0.649 to 0.989; k = 13), multiple instance learning frameworks 0.865 (range 0.770 to 0.920; k = 4), and vision transformers 0.860 (range 0.728 to 0.954; k = 3). The ordering, illustrated in Figure 6, places foundation models at the upper end of the performance spectrum, although the wide and overlapping ranges across all classes underscore the contribution of cohort composition, reference-standard quality and stain heterogeneity to observed accuracy.

The constituent studies of each subgroup are specified below to permit full reproducibility. The foundation-model subgroup (k = 19) comprised references [17, 35, 45, 52, 57, 61, 62, 63, 75, 79, 80, 82, 84, 88, 95, 98, 100, 106, 107], whilst the remaining 32 studies constituted the task-specific subgroup. Within the task-specific category, convolutional neural networks accounted for 13 studies [29, 38, 43, 46, 55, 59, 60, 74, 83, 87, 89, 93, 103], multiple instance learning frameworks for four [36, 65, 94, 102], vision transformers for three [51, 67, 68], and architecturally unspecified or hybrid deep-learning models for the remaining twelve [10, 30, 33, 34, 39, 40, 53, 64, 71, 97, 99, 105].

With respect to validation strategy, 10 studies reported internal validation only [33, 34, 38, 39, 43, 64, 67, 75, 103, 107], whilst the remaining 41 reported at least one externally validated cohort. With respect to publication status, 20 studies were preprints at the time of data extraction [35, 36, 38, 39, 45, 53, 55, 62, 64, 65, 67, 74, 75, 79, 93, 98, 100, 103, 106, 107], and the remaining 31 were peer-reviewed publications. Peer-reviewed studies (median AUC 0.894) and preprints (median AUC 0.910) did not differ significantly (Mann-Whitney U = 340.0, p = 0.569), indicating that the inclusion of preprint evidence did not materially bias the synthesis.

**Figure 7:**
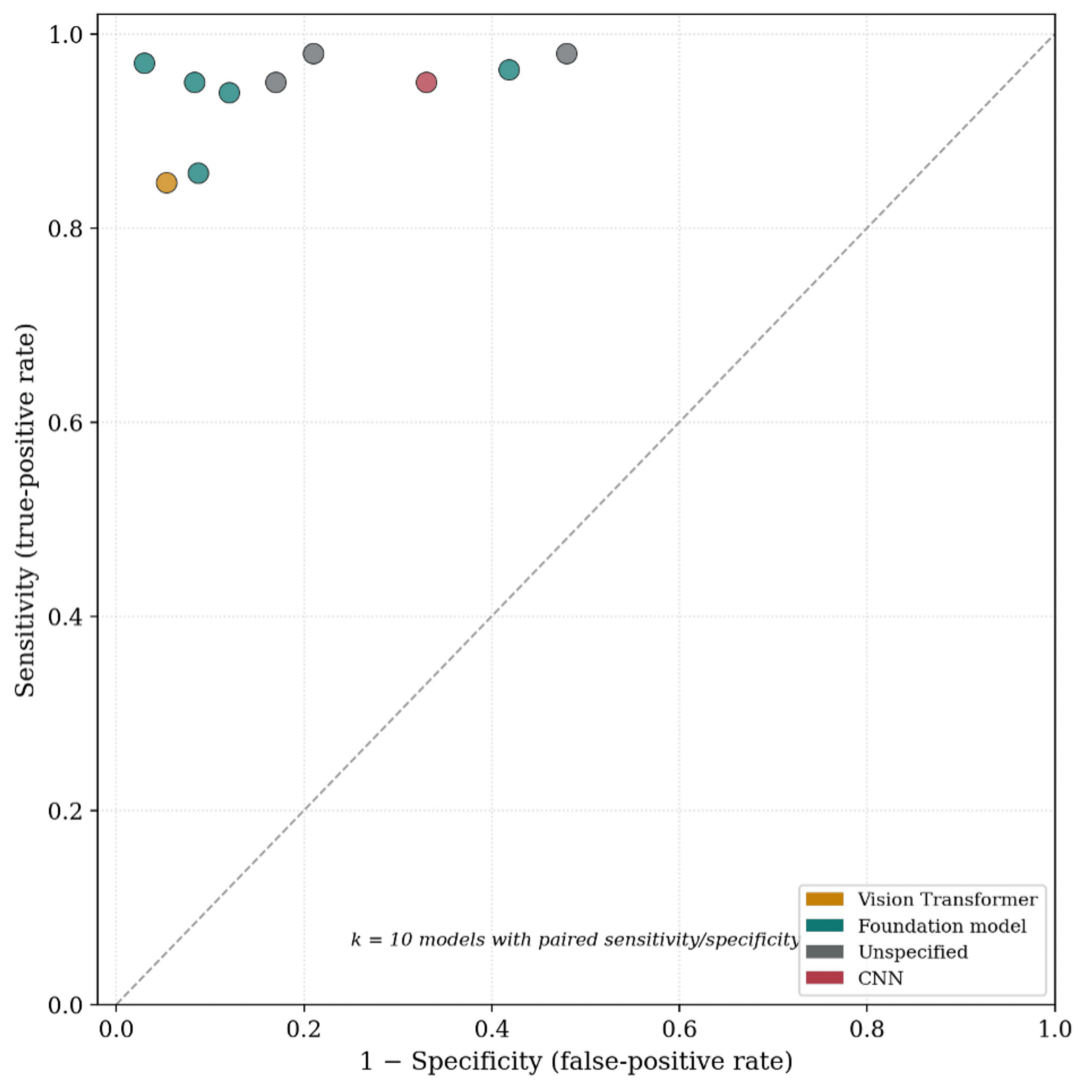
Reported sensitivity and specificity pairs for the ten studies providing paired operating-point data, plotted in ROC space and coloured by model class The diagonal denotes the line of no discrimination. ROC: receiver operating characteristic.

Ten studies reported a paired sensitivity and specificity at a defined operating threshold, permitting placement in receiver operating characteristic space (Figure 7). These estimates clustered in the upper-left quadrant of the ROC plane, consistent with the high discriminative performance reflected by the AUC synthesis. The small number of studies reporting reconstructable two-by-two contingency data precluded a formal bivariate (Reitsma) random-effects model or derivation of a hierarchical summary ROC curve at this stage; these analyses are deferred pending the receipt of contingency-table data requested from the corresponding authors of eligible studies.

Discussion

This systematic review and meta-analysis comprehensively synthesised the diagnostic accuracy of artificial intelligence models predicting microsatellite instability or mismatch-repair status directly from H&E-stained colorectal cancer histopathology, across 82 included studies of which 51 contributed extractable AUC data. The central finding is that contemporary models achieve consistently strong discrimination, with a median AUC of 0.895 and three-quarters of studies reporting an AUC at or above 0.79. This performance, achieved using only routinely stained tissue sections, supports the proposition first advanced by Kather et al. [9] that morphological correlates of the hypermutated phenotype are sufficiently expressed on H&E to permit reliable computational detection, and that such models may serve as an inexpensive triage step preceding confirmatory molecular testing [10].

A principal motivation for this updated review was the rapid emergence of histopathology foundation models trained by self-supervised learning on millions of slides [13-16]. In the present synthesis, foundation-model approaches achieved a higher median AUC than task-specific architectures (0.910 vs 0.879), with a narrower interquartile range suggesting more consistent performance. Although this difference did not reach statistical significance (U = 383.5, p = 0.124), the direction and consistency of the effect are concordant with the broader computational-pathology literature, in which general-purpose encoders such as UNI, Virchow, Prov-GigaPath and CTransPath have repeatedly improved label efficiency and cross-domain transfer. The earlier qualitative review by Park et al. [18] anticipated this trajectory, and the recent deep-learning meta-analysis by Li et al. [88] reported similar central tendencies for whole-slide deep-learning approaches; the present review extends these observations by explicitly contrasting foundation-model and task-specific architectures within an updated cohort that includes the post-2024 generation of pathology foundation models. The concordance of central tendencies across independent cohorts, and across the present and prior reviews, is most plausibly explained by the biological robustness of the underlying phenotype. Microsatellite-unstable tumours share a reproducible constellation of morphological features - dense tumour-infiltrating lymphocytes, poor differentiation, mucinous or medullary architecture, a Crohn-like peritumoural reaction and an expansile growth pattern - that is consistently expressed on haematoxylin and eosin and is therefore learnable by diverse model architectures trained on different cohorts. Three considerations temper this interpretation, however. First, a substantial proportion of studies draw upon overlapping public repositories, most notably The Cancer Genome Atlas, such that apparent cross-study agreement partly reflects shared data provenance rather than fully independent replication. Second, methodological convergence - towards weakly supervised multiple-instance-learning pipelines, stain normalisation and shared foundation-model encoders - homogenises both approach and output. Third, the area under the receiver operating characteristic curve is threshold-independent, intrinsically high for a well-separated binary phenotype, and preferentially reported, which may narrow the apparent spread of performance.

A notable and somewhat unexpected observation was that externally validated models performed almost identically to internally validated ones (0.895 vs 0.893; U = 224.5, p = 0.652). The diagnostic-accuracy literature more typically documents a fall in performance upon external validation, attributable to domain shift arising from scanner, staining and population differences. Two interpretations merit consideration. First, modern architectures, combined with stain-normalisation pipelines and large heterogeneous training corpora, may genuinely have narrowed the generalisation gap. Second, the inconsistent definition and reporting of external validation across studies may have inflated the apparent robustness; some studies designated as externally validated relied on cohorts that shared substantial methodological provenance with their development data. Standardised, geographically independent validation remains essential before these tools can be considered deployment-ready.

Substantial heterogeneity was evident across included studies, reflecting variation in reference-standard modality, cohort source, magnification, stain-normalisation strategy and scanner platform. A recurring methodological limitation, also evident in the QUADAS-2 risk-of-bias evaluation (Figure 2), was the inconsistent reporting of reconstructable two-by-two contingency data: only 10% of studies were judged at low risk in the index test domain, principally because a single discrimination metric was reported without a prespecified classification threshold or paired sensitivity-specificity. This reporting deficit currently precludes a fully specified bivariate random-effects model and hierarchical summary ROC curve, and constitutes the single most important barrier to quantitative evidence synthesis in this field. The reassuring absence of a significant difference between peer-reviewed articles and preprints suggests that the inclusion of high-quality preprints broadened coverage without introducing systematic bias.

The clinical promise of H&E-based MSI prediction lies in pre-screening: an accurate, inexpensive model could prioritise tumours for confirmatory immunohistochemistry or molecular testing, reduce reagent and turnaround burdens, and extend biomarker-informed care to settings where universal molecular testing is not feasible. The CE-IVD marking of a dedicated pre-screening tool [17] demonstrates that regulatory-grade translation is achievable. However, a screening role demands a deliberate emphasis on sensitivity to avoid missing immunotherapy-eligible patients, and any deployment must be accompanied by prospective validation, calibration assessment, and clear governance regarding the handling of indeterminate predictions.

Limitations of the Study

Several limitations merit acknowledgement. First, the quantitative synthesis was descriptive rather than model-based, owing to the limited availability of reconstructable two-by-two contingency data and reported confidence intervals across included studies. Pooled estimates therefore do not incorporate study-level precision weighting, and the bivariate random-effects Reitsma model with hierarchical summary ROC curve and formal Deeks publication-bias testing has been deferred pending the receipt of contingency-table data from study authors. Second, data extraction was initially performed using automated full-text parsing of open-access journal-article XML and preprint PDFs, with subsequent dual-reviewer manual verification; nonetheless, the possibility of residual extraction error in fields reported only within figures or supplementary tables cannot be excluded. The risk-of-bias judgements presented in Figure 2 were derived using prespecified rules applied to the structured extraction workbook; the formal dual-reviewer item-level QUADAS-2 and QUADAS-AI assessment will be reported in the final version of this review. Third, restriction to English-language reports may have introduced language bias. Fourth, although Unpaywall queries identified open-access versions for the majority of paywalled records, 14 records remained inaccessible through programmatic retrieval and were obtained manually; 19 paywalled studies could not be retrieved and contributed only metadata to the synthesis. Finally, the rapidly evolving nature of the field means that further eligible studies will have appeared during the conduct of the review; an update search will be performed within four weeks of submission.

Future Directions

Future primary studies should adhere to AI-specific reporting standards (STARD-AI, TRIPOD+AI), report complete contingency tables at prespecified thresholds, and undertake prospective, geographically independent external validation with explicit calibration analysis. Head-to-head benchmarking of foundation models against task-specific architectures on shared public cohorts would clarify the magnitude of any genuine performance advantage. From a synthesis perspective, the acquisition of two-by-two contingency data from study authors will permit the bivariate and hierarchical summary ROC analyses, meta-regression of heterogeneity sources, formal Deeks publication-bias testing, and full GRADE certainty rating that the present descriptive synthesis necessarily defers. Standardised reporting frameworks for AI diagnostic studies, integration with prospective clinical-deployment trials, and the development of decision-curve analyses tailored to pre-screening applications represent the principal research priorities to enable confident clinical translation.

## Conclusions

Artificial intelligence applied to routine H&E-stained histopathology predicts microsatellite instability and mismatch-repair status in colorectal cancer with consistently high discriminative accuracy, and emerging histopathology foundation models show a promising, if not yet statistically confirmed, performance advantage over task-specific architectures. The comparability of externally and internally validated estimates is encouraging, but warrants cautious interpretation given heterogeneous validation practices.

Before these models can be translated into routine diagnostic pathways, the field must address persistent heterogeneity in reference standards, cohorts and reporting, and in particular the scarcity of reconstructable diagnostic-accuracy data that currently constrains formal meta-analysis. Standardised reporting, complete contingency-table disclosure, and prospective external validation with calibration assessment are the essential next steps. With these in place, H&E-based artificial-intelligence pre-screening has the potential to widen equitable access to biomarker-informed colorectal cancer care, particularly in resource-constrained settings.

## Appendices

Complete search strategy and analytical environment

This file provides the complete, executable search strings for every bibliographic database and preprint server interrogated, together with the analytical environment and software used. It supplements the condensed search-strategy description given in the main text. Boolean operators combined Medical Subject Headings, Emtree terms and free-text terms across four conceptual blocks - colorectal cancer; microsatellite instability or mismatch repair; artificial intelligence, deep learning or machine learning; and haematoxylin and eosin histopathology. Results were limited to English-language records.

**Table 7:** Complete search strings by source, with applied filters MeSH: Medical Subject Headings; tiab: title/abstract field; ti,ab,kw: title, abstract and keyword fields; TS: topic field; PPR: preprints source code in Europe PMC; WSI: whole-slide image; MSI: microsatellite instability; MMR: mismatch repair; CRC: colorectal cancer. Free-text synonym sets were applied within each conceptual block; database-native syntax was adapted from the common four-concept structure shown above.

Database	Complete search string	Applied filters
PubMed / MEDLINE	("Colorectal Neoplasms"[MeSH] OR "colorectal cancer"[tiab] OR "colorectal carcinoma"[tiab] OR "colon cancer"[tiab] OR "rectal cancer"[tiab] OR CRC[tiab]) AND ("Microsatellite Instability"[MeSH] OR "DNA Mismatch Repair"[MeSH] OR "microsatellite instability"[tiab] OR MSI[tiab] OR "MSI-H"[tiab] OR "mismatch repair"[tiab] OR MMR[tiab] OR dMMR[tiab]) AND ("Deep Learning"[MeSH] OR "deep learning"[tiab] OR "machine learning"[tiab] OR "artificial intelligence"[tiab] OR "convolutional neural network"[tiab] OR "neural network"[tiab] OR "computational pathology"[tiab] OR "foundation model"[tiab]) AND ("histopathology"[tiab] OR "whole slide"[tiab] OR "whole-slide image"[tiab] OR WSI[tiab] OR "haematoxylin and eosin"[tiab] OR "hematoxylin and eosin"[tiab] OR "H&E"[tiab])	English; humans; Jan 2018 – Dec 2026
Embase (Ovid / Emtree)	('colorectal tumor'/exp OR 'colorectal cancer':ti,ab,kw OR CRC:ti,ab,kw) AND ('microsatellite instability'/exp OR MSI:ti,ab,kw OR 'mismatch repair':ti,ab,kw OR MMR:ti,ab,kw OR dMMR:ti,ab,kw) AND ('deep learning'/exp OR 'machine learning'/exp OR 'artificial intelligence'/exp OR 'convolutional neural network':ti,ab,kw OR 'foundation model':ti,ab,kw) AND ('histopathology':ti,ab,kw OR 'whole slide':ti,ab,kw OR WSI:ti,ab,kw OR 'hematoxylin eosin':ti,ab,kw)	English; 2018–2026; reviews excluded
Scopus	TITLE-ABS-KEY(("colorectal cancer" OR "colorectal carcinoma" OR CRC) AND ("microsatellite instability" OR MSI OR "mismatch repair" OR MMR OR dMMR) AND ("deep learning" OR "machine learning" OR "artificial intelligence" OR "convolutional neural network" OR "foundation model") AND (histopathology OR "whole slide" OR WSI OR "hematoxylin and eosin")) AND PUBYEAR > 2017 AND PUBYEAR < 2027 AND (LIMIT-TO(LANGUAGE,"English")) AND (EXCLUDE(DOCTYPE,"re"))	English; 2018–2026; reviews excluded
Web of Science (Core Collection)	TS=(("colorectal cancer" OR "colorectal carcinoma" OR CRC) AND ("microsatellite instability" OR MSI OR "mismatch repair" OR MMR OR dMMR) AND ("deep learning" OR "machine learning" OR "artificial intelligence" OR "convolutional neural network" OR "foundation model") AND (histopathology OR "whole slide" OR WSI OR "hematoxylin and eosin"))	English; 2018–2026; document type ≠ Review
IEEE Xplore	("All Metadata":"colorectal" AND "All Metadata":"microsatellite" AND "All Metadata":("deep learning" OR "machine learning" OR "neural network"))	English; 2018–2026; Conferences + Journals
Europe PMC (preprints)	(colorectal AND (MSI OR "mismatch repair" OR MMR OR "microsatellite instability") AND ("deep learning" OR "machine learning" OR "artificial intelligence") AND (histopathology OR "whole slide" OR "H&E")) AND SRC:"PPR"	Preprint source filter (indexes bioRxiv, medRxiv, ResearchSquare, Preprints.org)
arXiv	(ti:"colorectal" OR abs:"colorectal") AND abs:("microsatellite" OR MSI OR MMR OR "mismatch repair") AND abs:("deep learning" OR "neural" OR transformer OR "machine learning")	Categories cs.CV, eess.IV; relevance sort

Analytical environment and software

All analyses were conducted in R version 4.x (R Foundation for Statistical Computing, Vienna, Austria), with independent cross-verification in Stata version 18 (StataCorp LLC, College Station, Texas, USA). The pooled area under the receiver operating characteristic curve was summarised as the median with interquartile range and full range. Between-subgroup comparisons were performed using the Mann-Whitney U test. Small-study effects were assessed by an Egger-style linear regression of the area under the curve against study precision, defined as the reciprocal of the square root of the sample size. All figures were generated within the same environment. The full analysis code and the complete list of package versions are available from the corresponding author on reasonable request.

Search execution and deduplication. Records retrieved from all seven sources were collated, and six cross-database duplicates were removed prior to title and abstract screening. Manual reference checks of the included articles and of prior systematic reviews were additionally conducted to identify further eligible studies. Open-access versions of paywalled records were sought programmatically via Unpaywall, with records inaccessible through automated retrieval obtained manually where possible.

Risk-of-bias assessment: domain-derivation rules and per-study judgements

This file provides the prespecified rules used to derive each risk-of-bias domain judgement from the structured data-extraction workbook, together with the per-study judgements for all 82 included studies across the four QUADAS-2 domains and the overall judgement. The overall judgement for each study was taken as the least favourable rating across the four domains, such that a single high-risk domain rendered the overall judgement high risk. A full dual-reviewer, item-level QUADAS-2 and QUADAS-AI assessment, incorporating the artificial-intelligence-specific signalling questions (data quality, annotation methodology, model development, missing-data handling, threshold determination, calibration and external validation), will accompany the final version of this review.

**Table 8:** Prespecified rules for deriving each risk-of-bias domain judgement

Domain	Low risk	Some concerns	High risk
D1: Patient selection	External validation + ≥3 cohort sources OR ≥2 cohort sources	TCGA-only or single cohort	No cohort source reported
D2: Index test	Architecture + threshold-specific sensitivity AND specificity both reported	Architecture reported but threshold-specific sens/spec not paired	Architecture vague OR extraction confidence LOW
D3: Reference standard	Multiple modalities (e.g., PCR + IHC, or PCR/IHC/NGS)	Single modality	No reference standard reported
D4: Flow & timing	External validation AND clear validation strategy	Internal validation only	Validation strategy unclear/not reported
Overall	All 4 domains LOW	At least one SOME (no HIGH)	Any domain HIGH

**Table 9:** Per-study QUADAS-2 risk-of-bias judgements for all 82 included studies D1–D4: the four QUADAS-2 risk-of-bias domains. Study labels follow the surname-and-year convention of the main-text reference list; where two studies share a surname and year, a suffix letter is appended. QUADAS-2: Quality Assessment of Diagnostic Accuracy Studies, version 2.

Study	D1: Patient selection	D2: Index test	D3: Reference standard	D4: Flow & timing	Overall
Echle 2020	Low	High	Low	Low	High
Cao 2020	Low	Some concerns	Low	Low	Some concerns
Bustos 2021	Some concerns	High	Low	Low	High
Lea 2021	Some concerns	High	Low	Low	High
Nguyen 2021	Low	High	Low	High	High
Neumann 2021	High	High	Some concerns	Some concerns	High
Bilal 2021	Low	High	Low	Low	High
Mittal 2021	High	High	Low	Some concerns	High
Charlie 2021	Low	Some concerns	Low	Low	Some concerns
Yoni 2021	Some concerns	Some concerns	Low	Low	Some concerns
Xu 2021	Some concerns	High	Some concerns	High	High
Ruqayya 2022	Low	Some concerns	Low	Some concerns	Some concerns
Bustos 2022	Some concerns	High	Low	Some concerns	High
Xu 2022	Some concerns	High	Some concerns	High	High
Hyung 2022	Some concerns	High	Low	Low	High
Leiby 2022	Low	High	High	Low	High
Saldanha 2022	Low	Some concerns	Low	Low	Some concerns
Daniel 2022 (a)	Low	High	High	Some concerns	High
Saillard 2022	Low	Low	Low	Low	Low
Daniel 2022 (b)	Low	High	High	High	High
Qiu 2022	Low	Some concerns	High	Some concerns	High
Jiang 2022	Low	High	Low	Low	High
Guo 2023	Low	Low	Low	Low	Low
Min 2023 (a)	Low	Some concerns	Low	Low	Some concerns
Min 2023 (b)	Low	High	High	Low	High
Saillard 2023	Low	Some concerns	Low	Low	Some concerns
Kim 2023	Low	High	Low	Low	High
Alexander 2023	Low	High	High	Some concerns	High
Lafarge 2023	Low	Some concerns	Some concerns	Low	Some concerns
Niehues 2023	Low	Low	Low	Low	Low
Omar 2023	Low	Some concerns	High	Low	High
Tsai 2023	Low	Some concerns	Low	Low	Some concerns
Rubinstein 2023	Some concerns	Some concerns	Low	Some concerns	Some concerns
Sophia 2023	Low	Some concerns	Low	Low	Some concerns
Wagner 2023	Low	Some concerns	Low	Low	Some concerns
Chang 2023	Low	Low	Low	Low	Low
Tong 2023	Some concerns	Some concerns	Low	Low	Some concerns
Wu 2024	Some concerns	High	Some concerns	High	High
Lo 2024	High	Some concerns	Low	Low	High
He 2024	Low	High	Low	Some concerns	High
Hadar 2024	Low	Some concerns	Some concerns	Low	Some concerns
Kim 2024	Low	High	Low	High	High
Mao 2024	Some concerns	High	Low	High	High
Quan 2024	High	Some concerns	Some concerns	Some concerns	High
Loeffler 2024	Low	Some concerns	High	Low	High
Gustav 2024	Low	Some concerns	Low	Low	Some concerns
Sinicrope 2024	Some concerns	High	Low	Low	High
Xiaojian 2024	Low	Some concerns	Low	Some concerns	Some concerns
Zhang 2024	Low	Low	Low	Low	Low
Žigutytė 2024	Low	Some concerns	Some concerns	Some concerns	Some concerns
Aswolinskiy 2025	Low	Some concerns	Low	Low	Some concerns
Baumann 2025 (a)	Low	Some concerns	Low	Low	Some concerns
Luo 2025	Low	High	High	High	High
Buvaneswari 2025	High	High	High	High	High
Bass 2025	Low	High	Low	Some concerns	High
Myles 2025	Low	Some concerns	Low	Low	Some concerns
Zeng 2025	High	High	Some concerns	Low	High
El 2025	Some concerns	Some concerns	Some concerns	Low	Some concerns
Baumann 2025 (b)	Low	High	Some concerns	High	High
Wei 2025	Low	High	Low	Low	High
Li 2025	Low	Low	Low	Low	Low
Lee 2025	Low	Some concerns	Low	Low	Some concerns
Millward 2025	Low	High	Some concerns	Some concerns	High
Bian 2025	Some concerns	Some concerns	Low	Low	Some concerns
Piotr 2025	Low	Some concerns	Some concerns	Low	Some concerns
Liu 2025	Some concerns	Some concerns	Low	Low	Some concerns
Gustav 2025	Low	Some concerns	Some concerns	Low	Some concerns
Trahearn 2025	High	Some concerns	Some concerns	Some concerns	High
Petäinen 2025	Low	Some concerns	Low	Low	Some concerns
Yang 2025	High	High	Some concerns	Low	High
Shi 2025	Low	Low	High	Low	High
Shen 2025	Low	Some concerns	Some concerns	Low	Some concerns
Feng 2025	Low	Low	Low	Low	Low
Xu 2025	Some concerns	Some concerns	Low	Low	Some concerns
Zhang 2025	Some concerns	Some concerns	High	Low	High
Cheng 2025	Low	Some concerns	Low	Low	Some concerns
Zamanitajeddin 2025	Low	Some concerns	Low	Low	Some concerns
Huang 2026	Low	Some concerns	Low	Some concerns	Some concerns
Li 2026	High	High	Low	Low	High
Koh 2026	High	High	Low	Some concerns	High
Dasari 2026	Low	Some concerns	Low	Low	Some concerns
Lydia 2026	Low	Some concerns	Low	Some concerns	Some concerns
